# Efficient One‐Pot Synthesis of Benzothiazole Compounds From Vinamidinium Salts

**DOI:** 10.1002/open.202500540

**Published:** 2025-12-06

**Authors:** Zeinab khosravi, Abdolmohammad Mehranpour, Mohammad Reza Mohammadizadeh, Genta Kojya, Satoru Arimitsuc

**Affiliations:** ^1^ Department of Chemistry Faculty of Nano and Bio Science and Technology Persian Gulf University Bushehr Iran; ^2^ Center for Research Advancement and Collaboration University of the Ryukyus Nishihara Japan; ^3^ Graduate School of Engineering and Science University of the Ryukyus Nishihara Japan

**Keywords:** benzothiazole, Schiff base, triethyl amin, vinamidinium salt

## Abstract

In this study, the condensation reaction between vinamidinium salts and Z‐2‐hydrazineylidene‐3‐methyl‐2,3‐dihydrobenzo[d]thiazole is described for the synthesis of 1,3‐bis((3‐methylbenzo[d]thiazol‐2(3H)‐ylidene)hydrazineylidene)propan‐2‐ol (BBTA) or 1‐((1Z,3E)‐1‐(dimethylamino)‐3‐(((Z)‐3‐methylbenzo[d]thiazol‐2(3H)‐ylidene)hydrazineylidene)prop‐1‐en‐2‐yl)pyridin‐1‐ium (DMBT). A noteworthy aspect of the synthesis of these derivatives is that the structure of the vinamidinium salt acts as the determining factor for the type of product formed. Specifically, phenyl‐substituted vinamidinium salts predominantly lead to the formation of symmetric BBTA derivatives, whereas pyridinium‐based vinamidinium salts favor the production of asymmetric DMBT compounds. The reaction was carried out in the presence of triethylamine as the base and acetonitrile as the solvent under catalyst‐free conditions. The structure of the new products was confirmed based on their spectral data, including ^1^H NMR, ^13^C NMR, IR, mass spectrometry, high‐resolution mass spectrometry and X‐ray analysis.

## Graphical abstract

A catalyst‐free one‐pot reaction of vinamidinium salts with benzothiazole hydrazines efficiently produces 1,3‐bis((3‐methylbenzo[d]thiazol‐2(3H)‐ylidene)hydrazineylidene)propan‐2‐ol (BBTA) and 1‐((1Z,3E)‐1‐(dimethylamino)‐3‐(((Z)‐3‐methylbenzo[d]thiazol‐2(3H)‐ylidene)hydrazineylidene)prop‐1‐en‐2‐yl)pyridin‐1‐ium (DMBT) derivatives.This simple method enables rapid access to diverse benzothiazole frameworks.

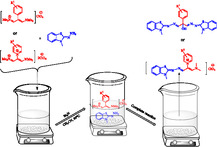

## 1 Introduction

One of the most valuable intermediates in the synthesis of organic molecules is the three‐carbon compound known as a vinamidinium salt [1, 2, 3, 4, 5, 6, 7, 8]. In 1968, Arnold utilized the Vilsmeier reagent to react carboxylic acids containing two hydrogen atoms at the alpha carbonyl position, leading to the creation of the first vinamidinium salts [9, 10, 11, 12]. The Vilsmeier–Haack conditions remain the most commonly employed method for synthesizing this type of salt [13]. Similar to malonaldehydes [14], vinamidinium salts undergo a potential condensation process so that when identical condensation reactions occur with vinamidinium salts, dimethylamine is generated as a byproduct instead of H_2_O molecules [15, 16, 17, 18]. One of the distinguished characteristics of vinamidinium salts is that the happening resonance between the electron donor and acceptor groups within the molecule contributes to the sufficient stability of these salts [19, 20, 21, 22, 23]. In particular, this characteristic strengthen their reactivity toward dinucleophilic reagents, which make them as powerful building blocks for the production of different molecules [24, 25]. Due to their basic components, vinamidinium salts are not only used in medical applications but also as pigments for producing specific colors, further emphasizing the importance of their synthesis [26]. Singh et al. utilized Schiff‐base ligands with the target products as the high flexibility of these ligands, in which case allows them to form complexes with various metal ions, exhibiting the desired properties [27]. Some of these Schiff‐base complexes are known to display antifungal and anticancer activities, making them important biological species [28, 29]. Furthermore, these types of complexes are valuable for industrial applications due to their straightforward synthesis and the available of low‐cost reagents [30, 31, 32].

Chemists have focused on the synthesis of heterocyclic compounds, particularly those containing sulfur and nitrogen owing to their importance in the development of pharmaceuticals and insecticides [33]. Among these, thiazoles are highly valued for their therapeutic properties [34]. By reason of their significant biological activities, including antibacterial [35], antimalarial [36], antiallergic [37], anti‐inflammatory [38], antihypertensive [39], and anti‐HIV properties [40], thiazole and its derivatives have also shown remarkable potential in the treatment of tumors [41] and cancer [42]. These unique properties make thiazole an ideal target for synthesis, especially when the synthesis process is simple, efficient, and highly effective.

In this study, we synthesized heterocyclic compounds with a benzothiazole skeleton via Wiles–Meier reactions and vinamidinium salts using readily available and low‐cost reagents through a simple and one‐pot approach. The results demonstrated high efficiency. The following substances were used in this study: Z‐2‐hydrazineylidene‐3‐methyl‐2,3‐dihydrobenzo[d]thiazole, vinamidinium salts, and triethylamine as the base, under reflux conditions with acetonitrile as the solvent. The significance of our study lies in the fact that this method enables the efficient synthesis of both symmetric and asymmetric bulky compounds using inexpensive, readily available starting materials and straightforward, practical methods without the use of catalysts from vinamidinium salts.

## 2 Experimental

### 2.1 Materials and Apparatuses

2‐Hydrazinylidine‐3‐methyl‐2,3‐dihydrobenzo[d]thiazole compounds, vinamidinium salts, and triethylamine were commercially available. All additional reagents and solvents were purchased commercially and purified according to standard methods.

^1^H NMR (300 MHz) and ^13^C NMR (75 MHz) spectra were run on Bruker in pure deuterated dimethylsulfoxide (DMSO‐*d*_6_), chloroform (CDCl_3_), and acetonitrile (CD_3_CN).

Chemical shifts are given in the δ scale in part per million (ppm) and *J* in Hz. Data for ^1^H NMR are reported as follows: chemical shift (δ ppm), multiplicity (s = singlet, d = doublet, t = triplet, q = quartet, m = multiplet, and dd =  doublet of doublets), coupling constant (Hz), and integration. Melting points were determined in open capillary tubes in a Buchi‐510 oil melting point apparatus.

### 2.2 The General Method of BBTA and DMBT Synthesis (Scheme 1)

**SCHEME 1:**
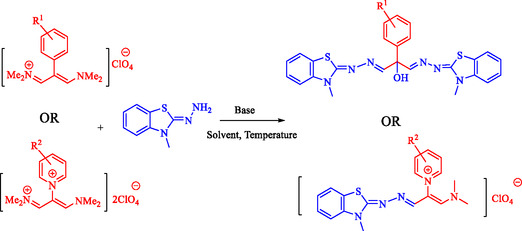
Synthesis of BBTA and DMBT.

A mixture of 2‐hydrazinylidene‐3‐methyl‐2,3‐dihydrobenzo[d]thiazole (1.0 for pyridine derivatives or 2.0 mmol for phenyl derivatives), vinamidinium salts (1.0 mmol), and triethylamine (0.5 mL) as the base in 5 mL of acetonitrile solvent was stirred at 80°C for 12 h. After cooling, water was added, and the formed precipitate was filtered and recrystallized in ethanol. The BBTA and DMBT product was obtained with high efficiency, yielding over 83% (Scheme 1).

**SCHEME 2:**
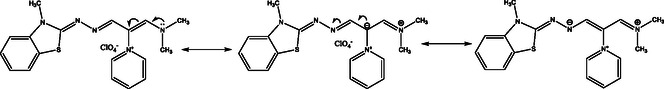
Preferred resonance structures for DMBT.

Analysis of the spectral data of some products containing pyridine salts (DMBT derivatives) not only confirms this structure but also reveals an interesting phenomenon: due to the significant resonance present at both ends of the polyene chain in the DMBT compound (Scheme 2), a high degree of double‐bond character is established between the NMe_2_ group and its adjacent carbon. This phenomenon restricts rotation around the aforementioned bond, causing the methyl groups to experience distinct chemical environments. Consequently, two separate peaks, each with an integral of 3H (for a total integral of 6H), are observed in the range of δ 2–3 for the NMe_2_ protons (Scheme 2).

### 2.3 Selected Spectral Data of BBTA and DMBT

#### 2.3.1 1‐((1Z,3E)‐1‐(dimethylamino)‐3‐(((Z)‐3‐methylbenzo[d]thiazol‐2(3H)‐ylidene)hydrazineylidene)prop‐1‐en‐2‐yl)pyridin‐1‐ium (1a)

Crimson color powder (393 mg, 90%), m.p. 217–220°C, ^1^H NMR (DMSO‐*d*_6_, 300 MHz) δ (ppm): 2.66 (s, 3H, CH_3_), 2.74–2.81 (m, 3H, CH_3_), 3.22 (s, 2H, CH_3_), 3.46 (d, *J *= 9 Hz, 1H, CH_3_), 7.0–7.59 (m, 6H, Ar–H, and CH), 7.84–8.61 (m, 3H, Ar–H, and CH), 8.71–8.86 (m, 1H, Ar–H), 9.14–9.20 (m, 1H, Ar–H). ^13^C NMR (DMSO‐*d*_6_, 75 MHz) δ (ppm): 31, 42.4, 44.2, 110, 110.9, 121.8, 122.7, 126.7, 127.4, 127.7, 143.9, 146.2, 146.5, 146.9, 149.7, 164.4. MS m/z (%): 257(100), 302(45), 496(45).

#### 2.3.2 1‐((1Z,3E)‐1‐(dimethylamino)‐3‐(((Z)‐3‐methylbenzo[d]thiazol‐2(3H)‐ylidene)hydrazineylidene)prop‐1‐en‐2‐yl)‐3,5‐dimethylpyridin‐1‐ium (1b)

Crimson color powder (409 mg, 88%), m.p. 225–233°C, ^1^H NMR (CD_3_CN, 300 MHz) δ (ppm): 2.54–2.59 (m, 6H, CH_3_), 2.68–2.92 (m, 6H, CH_3_), 3.48 (d, *J* *=* 6 Hz, 3H, CH_3_), 6.86 (s, 1H, CH), 6.99–7.12 (m, 2H, Ar–H), 7.26–7.32 (m, 1H, Ar–H), 7.36–7.39 (m, 1H, Ar–H), 7.99 (s, 1H, CH), 8.28 (d, *J *= 21 Hz, 1H, Ar–H), 8.43–8.58 (m, 2H, Ar–H). ^13^C NMR (CD_3_CN, 75 MHz) δ (ppm): 17.9, 30.9, 42.5, 44.2, 109.8, 111.7, 117.8, 121.8, 122.4, 124.3, 126.8, 138.7, 142, 143.1, 145.9, 148.1, 150.3, 164.7. MS m/z (%): 161(65), 207(65), 241(73), 257(100), 355(60).

#### 2.3.3 1‐((1Z,3E)‐1‐(dimethylamino)‐3‐(((Z)‐3‐methylbenzo[d]thiazol‐2(3H)‐ylidene)hydrazineylidene)prop‐1‐en‐2‐yl)quinolin‐1‐ium (1c)

Dark brown powder (419 mg, 86%), m.p. 210–215°C, ^1^H NMR (DMSO‐*d*_6_, 300 MHz) δ (ppm): 2.62 (s, 6H, CH_3_), 3.26 (s, 1H, CH_3_), 3.39 (d, *J *= 6 Hz, 2H, CH_3_), 6.90–7.35 (m, 4H, Ar–H), 7.54 (s, 1H, CH), 8.08–8.69 (m, 6H, Ar–H, and CH), 9.53–9.64 (dd, *J *= 8.2 Hz, *J *= 5.6 Hz, 2H, Ar–H).

#### 2.3.4 2‐((1Z,3E)‐1‐(dimethylamino)‐3‐(((Z)‐3‐methylbenzo[d]thiazol‐2(3H)‐ylidene)hydrazineylidene)prop‐1‐en‐2‐yl)isoquinolin‐2‐ium (1d)

Dark brown powder (390 mg, 80%), m.p. > 230°C, ^1^H NMR (CD_3_CN, 300 MHz) δ (ppm): 2.18 (s, 6H, CH_3_), 2.78 (s, 3H, CH_3_), 6.95–7.29 (m, 5H, Ar–H, and CH), 8.10–8.68 (m, 7H, Ar–H, and CH), 9.57–9.68 (m, 1H, Ar–H).

#### 2.3.5 4‐(tert‐butyl)‐1‐((1Z,3E)‐1‐(dimethylamino)‐3‐(((Z)‐3‐methylbenzo[d]thiazol‐2(3H)‐ylidene)hydrazineylidene)prop‐1‐en‐2‐yl)pyridin‐1‐ium (1e)

Brown powder (409 mg, 83%), m.p. > 230°C, ^1^H NMR (DMSO‐*d*_6_, 300 MHz) δ (ppm): 1.39–1.47 (m, 9H, CH_3_), 2.64–3.18 (m, 6H, CH_3_), 3.20 (s, 1H, CH_3_), 3.45 (d, *J *= 10.9 Hz, 2H, CH_3_), 6.96–7.52 (m, 5H, Ar–H, and CH), 7.81–8.57 (m, 3H, Ar–H, and CH), 8.72–9.08 (m, 2H, Ar–H).

#### 2.3.6 (1E,3E)‐1,3‐bis(((Z)‐3‐methylbenzo[d]thiazol‐2(3H)‐ylidene)hydrazineylidene)‐2‐phenylpropan‐2‐ol (1f)

Crimson color powder (461 mg, 95%), m.p. 200–202°C, ^1^H NMR (CDCl_3_, 300 MHz) δ (ppm): 3.62 (s, 6H, CH_3_), 5.08 (s, 1H, OH), 7.04–7.13 (m, 4H, Ar–H), 7.32–7.46 (m, 7H, Ar–H), 7.69–7.71 (m, 2H, Ar–H), 8.22 (s, 2H, CH). ^13^C NMR (CDCl_3_, 75 MHz) δ (ppm): 31.3, 60.9, 109.6, 122.2, 124.4, 126.1, 126.4, 127.7, 128.5, 132.1, 140.8, 148.1, 156.9, 168.4. IR (KBr) ν: 3301, 2958, 1641, 1560, *λ*_max_ (DMSO) = 361.

#### 2.3.7 (1E,3E)‐2‐(4‐bromophenyl)‐1,3‐bis(((Z)‐3‐methylbenzo[d]thiazol‐2(3H)‐ylidene)hydrazineylidene)propan‐2‐ol (1g)

Crimson color powder (502 mg, 89%), m.p. 204°C, ^1^H NMR (CDCl_3_, 300 MHz) δ (ppm):) 3.59 (s, 6H, CH_3_), 5.11 (s, 1H, OH), 7.02–7.12 (m, 4H, Ar–H), 7.28–7.34 (m, 2H, Ar–H), 7.44 (d, *J* *=* 8.1 Hz, 2H, Ar–H) 7.52–7.61 (m, 4H, Ar–H), 8.14 (s, 2H, CH). ^13^C NMR (CDCl_3_, 75 MHz) δ (ppm): 30.9, 75.9, 109.3, 121.8, 122.1, 124.3, 126.2, 128, 131.5, 140.2, 140.9, 156.2, 168.4. *λ*_max_ (DMSO) = 363.

#### 2.3.8 (1E,3E)‐2‐(4‐chlorophenyl)‐1,3‐bis(((Z)‐3‐methylbenzo[d]thiazol‐2(3H)‐ylidene)hydrazineylidene)propan‐2‐ol (1h)

Crimson color powder (473 mg, 91%), m.p. 185–190°C, ^1^H NMR (CDCl_3_, 300 MHz) δ (ppm): 3.59 (s, 6H, CH_3_), 5.11 (s, 1H, OH), 7.02–7.13 (m, 4H, Ar–H), 7.28–7.45 (m, 6H, Ar–H), 7.62–766 (m, 2H, Ar–H), 8.15 (s, 2H, CH). ^13^C NMR (CDCl_3_, 75 MHz) δ (ppm): 30.9, 75.8, 109.3, 121.9, 122.1, 124.3, 126.2, 127.6, 128.5, 133.6, 139.5, 140.9, 156.2, 168.5. MS m/z (%): 239(100), 257(95), 157(35), 355(30). *λ*_max_ (DMSO) = 367.

## 3 Results and Discussion

This research began with the reaction of vinamidinium salts and 2‐hydrazinylidene‐3‐methyl‐2,3‐dihydrobenzo[d]thiazole in acetonitrile solvent with *i*‐Pr_2_NEt as the base at 80°C (Table 1, entry 1). Although the reaction proceeded, the yield was lower than expected. Subsequently, several bases, including NaOCH_3_, NaH, DBU, K_2_CO_3_, KOH, and Et_3_N were screened to improve the yield (Table 1, entries 2–7). Among the bases used, the highest yield was obtained in the presence of triethylamine (Table 1, entry 7). Optimization of solvents revealed that ethanol is a suitable solvent compared to others, but its lower yield compared to acetonitrile led us to discontinue the reaction with this solvent. Although the reaction time is shorter with ethanol, acetonitrile performs better under the same conditions (Table 1, entries 8–12). We then examined the temperature and found that an initial temperature of 80°C was optimal for the reaction (Table 1, entries 13–14). Therefore, the most suitable conditions were selected.

**TABLE 1:** Optimization of reaction parameters.

Entry	Solvent	Base	Temp (°C)	Time (h)	Yield b (%)
1	CH_3_CN	*i*‐Pr_2_NEt	80	24	59
2	CH_3_CN	NaOCH_3_	80	24	36
3	CH_3_CN	NaH	80	24	Trace
4	CH_3_CN	DBU	80	24	Trace
5	CH_3_CN	K_2_CO_3_	80	24	63
6	CH_3_CN	KOH	80	24	42
7	CH_3_CN	Et_3_N	80	12	90
8	EtOH	Et_3_N	80	12	73
9	MetOH	Et_3_N	50	24	57
10	DMF	Et_3_N	130	24	Trace
11	CH_3_CN	—	80	24	—
12	Toluene	Et_3_N	110	24	Trace
13	CH_3_CN	Et_3_N	50	24	43
14	CH_3_CN	Et_3_N	r.t	24	trace

**SCHEME 3:**
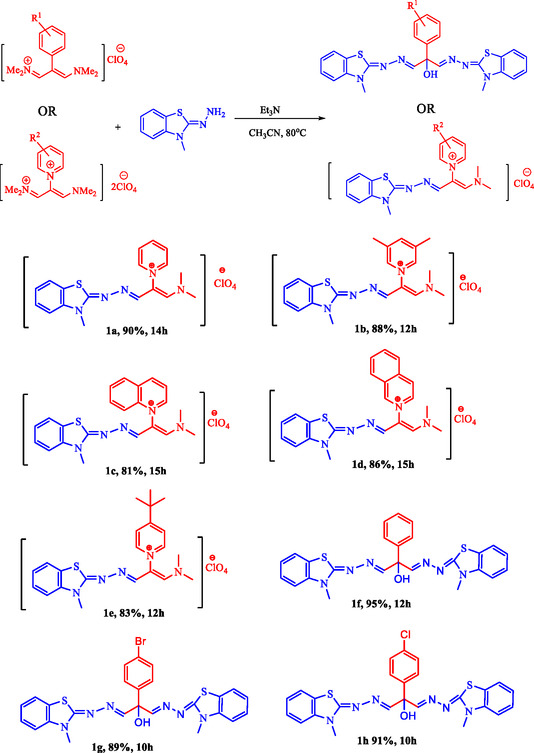
BBTA and DMBT derivatives.

Using the optimized conditions, BBTA and DMBT derivatives (1a–h) were synthesized, as shown in Scheme 3.

As shown in Scheme 3, pyridine derivatives increased the reaction time compared to phenyl derivatives, likely due to the instability of the positive charge on the nitrogen in the ring. The 3,5‐dimethyl donor group reduced the reaction time by stabilizing the positive charge on nitrogen (1b). On the other hand, although the tert‐butyl electron donor group also shortened the reaction time, its steric hindrance decreased the efficiency (1e). In phenyl derivatives, the electron‐withdrawing effects of chlorine and bromine atoms significantly reduced the reaction time compared to the phenyl derivative, while maintaining excellent efficiency (1g–1h).

**SCHEME 4:**
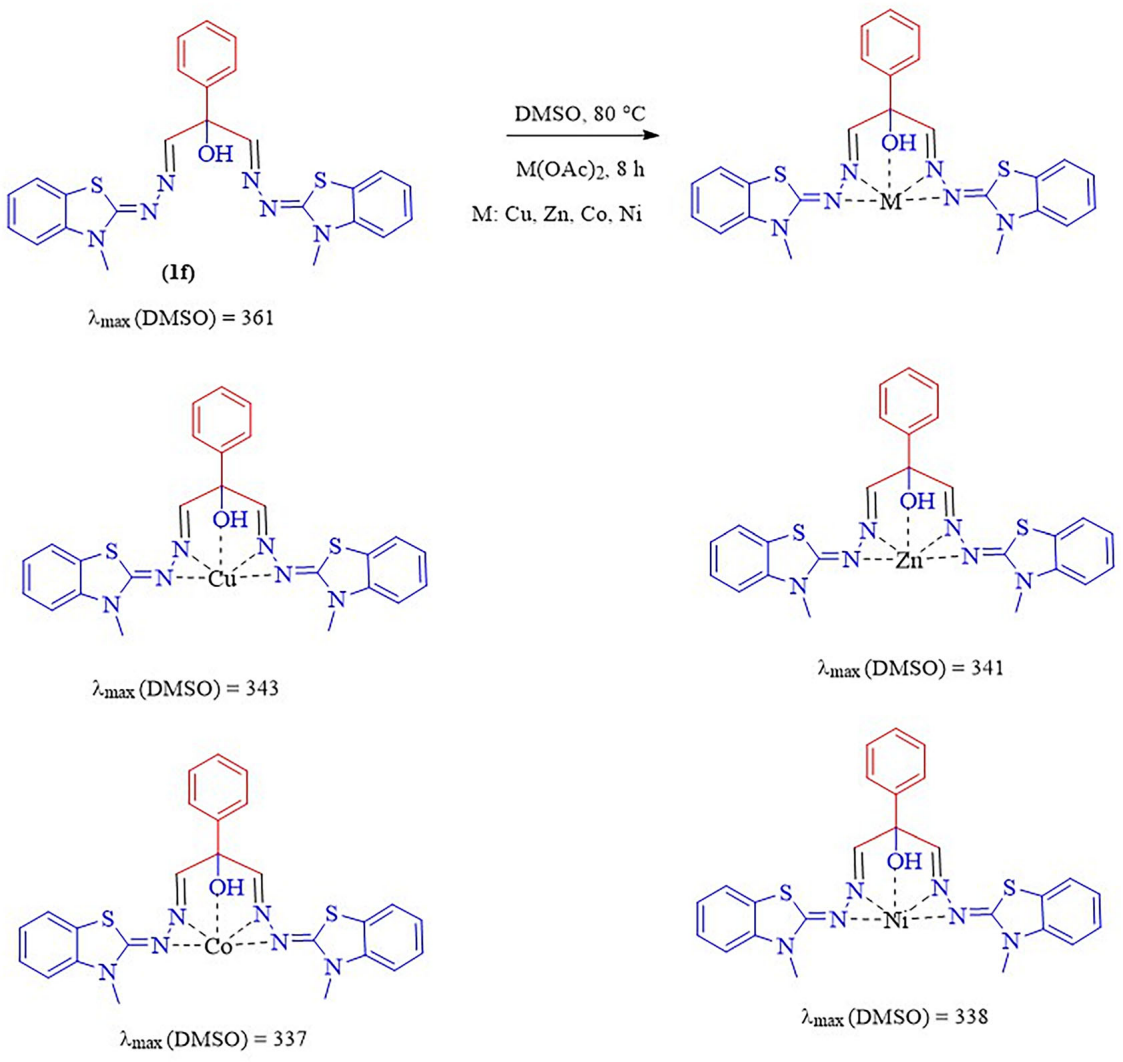
Complex formation between ligands and metals.

The formation of a base shift complex between oxygen and nitrogen ligands with metal ions in BBTA was investigated (Scheme 4). For this purpose, the phenyl derivative (1f) was ultrasonicated with nickel, cobalt, zinc, and copper acetate in DMSO solvent for 8 h. UV spectra were recorded before and after complex formation, as shown in Scheme 4. A decrease in *λ*_max was observed for all metals, indicating the formation of complexes.

**SCHEME 5:**
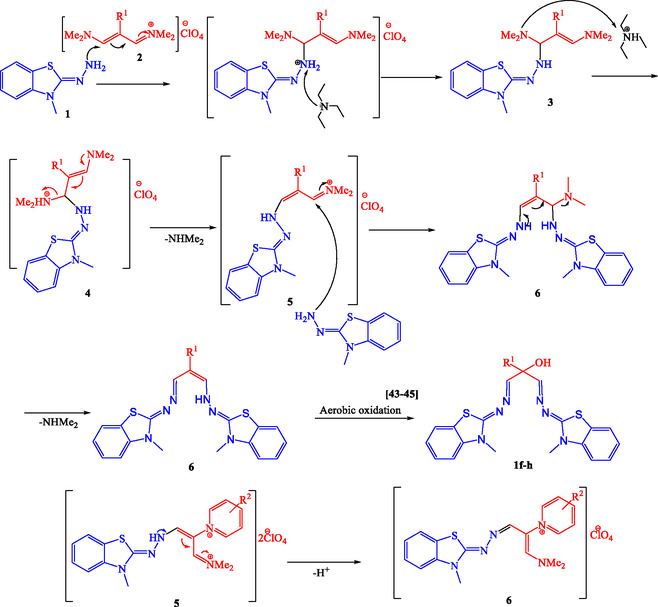
Proposed mechanism for the synthesis of BBTA and DMBT.

Although no control or kinetic reactions have been performed to prove the mechanism, based on the known chemistry of vinamidinium salts and benzothiazoles, the following mechanism is proposed for the preparation of the products( Scheme 5). First, intermediate **3** is formed by the nucleophilic attack of the deprotonated amine group in Z‐2‐hydrazineylidene‐3‐methyl‐2,3‐dihydrobenzo[d]thiazole **1** on the vinamidinium salts **2**. This is followed by the removal of dimethylamine and the nucleophilic attack of a second molecule of Z‐2‐hydrazineylidene‐3‐methyl‐2,3‐dihydrobenzo[d]thiazole on the resulting vinamidinium salt **5** to produce intermediate **6**. The loss of the second dimethylamine molecule in this step, followed by aerobic oxidation [43, 44, 45], leads to the formation of the desired products 1f–h. In step 5, the positive hydrogen is removed, resulting in the final product **7**, as seen for pyridine derivatives (DMBT). The positive charge on the ring nitrogen and the electron‐withdrawing nature of the second benzothiazole seem to prevent it from attacking the molecule in step 5. However, if an attack occurs, it typically returns to the stability of salt 1a–e.

**FIGURE 1:**
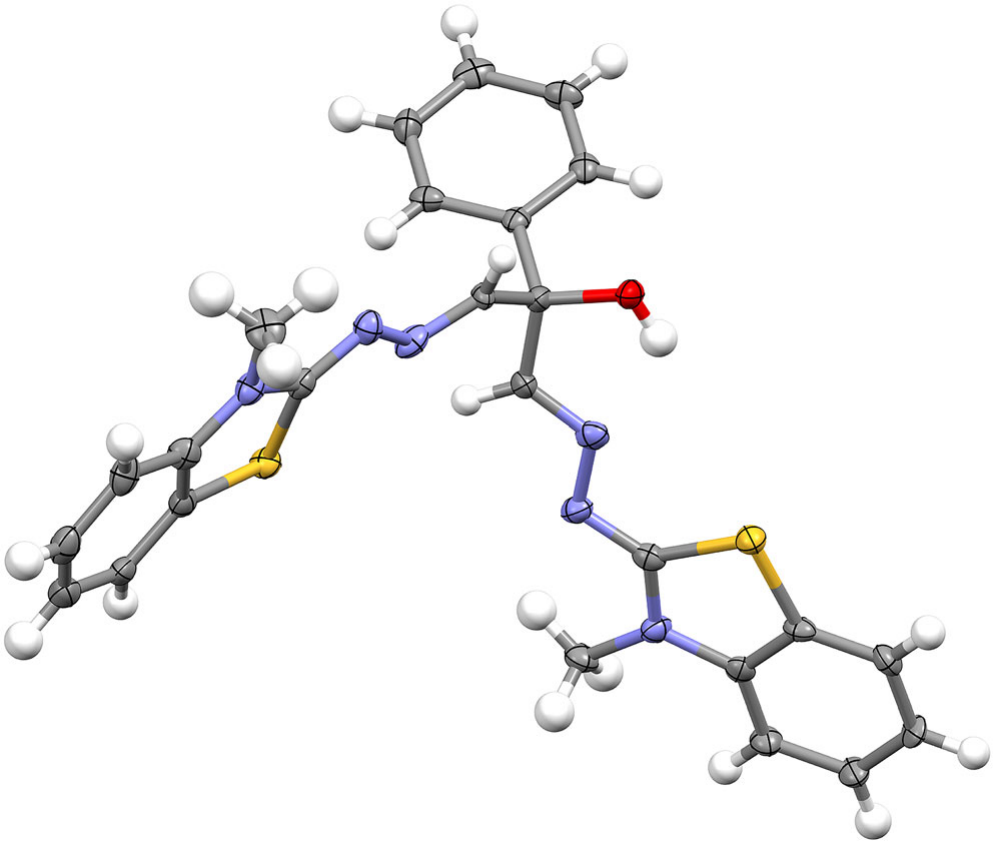
X‐ray structure of 1f.

As shown in Figure 1, X‐ray analysis was conducted to determine the structure of phenyl derivatives with greater precision. X‐ray data is provided in Supplementary Table 1f (S13). This table contains the CCDC reference code of the supplementary crystallographic data for this article (DOI: 10.5517/ccdc.csd.cc2j8zb4). These data can be obtained free of charge from The Cambridge Crystallographic Data Centre (http://www.ccdc.cam.ac.uk). One of the most significant van der Waals interactions in crystal formation is the π—π bond, which is seen in Supplementary Figures S15 and S16.

## 4 Conclusions

Briefly, we introduced, one‐pot approach for synthesizing benzothiazole compounds with several key benefits: 1) it is efficient, straightforward, and highly effective; 2) it does not require the application of a catalyst; 3) it enables the creation of both symmetric and asymmetric large compounds; and 4) it allows for complex formation with different metals. The results showed that different bases and solvent conditions significantly influenced reaction time and product yield. The use of electron‐donating groups, such as 3,5‐dimethyl, and resonance electron‐donating groups like chlorine and bromine in phenyl derivatives led to notable changes in reaction time and efficiency. Spectral techniques such as UV and X‐ray analysis were employed to accurately identify the structure and crystalline properties of the synthesized compounds. The findings indicated that van der Waals interactions, particularly π–π interactions, play a crucial role in crystal formation. This study demonstrates that careful selection of reaction conditions and thorough analysis of molecular properties can optimize the synthesis of target compounds and improve reaction yields.

## Supporting Information

Additional supporting information can be found online in the Supporting Information section. **Supporting** **Fig.** **S1:** The ^1^HNMR spectrum of compound **1a**. **Supporting** **Fig.** **S2:** The ^13^CNMR spectrum of compound **1a**. **Supporting** **Fig.** **S3:** The Mass spectrum of compound **1a**. **Supporting** **Fig.** **S4:** The ^1^HNMR spectrum of compound **1b**. **Supporting Fig. S5:** The ^13^C NMR spectrum of compound **1b**. **Supporting Fig. S6:** The Mass spectrum of compound **1b**. **Supporting Fig. S7:** The ^1^HNMR spectrum of compound **1c**. **Supporting Fig. S8:** The ^1^HNMR spectrum of compound **1d**. **Supporting Fig. S9:** The ^1^HNMR spectrum of compound **1e**. **Supporting Fig. S10:** The ^1^HNMR spectrum of compound **1f**. **Supporting Fig. S11:** The ^13^CNMR spectrum of compound **1f**. **Supporting Fig. S12:** The X‐RAY spectrum of compound **1f**. **Supporting Fig. S13:** The X‐RAY data of compound **1f**. **Supporting Fig. S14:** The High resolution Mass spectrum of compound **1f**. **Supporting Fig. S15:** The FT‐IR spectrum of compound **1f**. **Supporting Fig. S16:** The Vanderwals bonds of compound **1f**. **S** **upporting Fig. S17:** The π–π Bonds of compound **1f**. **Supporting Fig. S18:** The ^1^HNMR spectrum of compound **1g**. **Supporting Fig. S19:** The ^13^C NMR spectrum of compound **1g**. **Supporting Fig. S20:** The ^1^HNMR spectrum of compound **1h**. **Supporting Fig. S21:** The ^13^C NMR spectrum of compound **1h**. **Supporting Fig. S22:** The Mass spectrum of compound **1h**.

## Author Contributions

**Zeinab khosravi** performed the experiments, collected the data, and wrote the initial draft of the manuscript. **Abdolmohammad Mehranpour** supervised the project and performed the final editing of the manuscript. **Mohammad Reza Mohammadizadeh** also assisted in the analysis of spectral data. **Genta Kojya** and **Satoru Arimitsuc** contributed to the preparation and interpretation of the analyses.

## Funding

The authors have nothing to report.

## Ethics Statement

The authors have nothing to report.

## Conflict of Interest

The authors declare no conflicts of interest.

## Supporting information

Supplementary Material

## Data Availability

The data that support the findings of this study are available in the supplementary material of this article.
