# Supplementary material for: Efficient One‐Pot Synthesis of Benzothiazole Compounds From Vinamidinium Salts
Source: ChemistryOpen. 2025 Dec 6;15(4):e202500540. doi: 10.1002/open.202500540 (PMC13052193; doi:10.1002/open.202500540)
Supplement: Supplementary file 1 — Supplementary Material [file OPEN-15-e202500540-s001.pdf]

Supplementary material

Efficient One-Pot Synthesis of Benzothiazole Compounds from Vinamidinium Salts

Zeinab khosravi[1]. Abdolmohammad Mehranpour[1]. Mohammad Reza Mohammadizadeh[1]. Genta Kojya[2]. Satoru Arimitsuc[3]

[1] Department of Chemistry, Faculty of Nano and Bio Science and Technology, Persian Gulf University, Bushehr 75169, Iran

[2] Center for Research Advancement and Collaboration, University of the Ryukyus, Senbaru 1, Nishihara, 903-0213, Okinawa, Japan

[3] Graduate School of Engineering and Science, University of the Ryukyus, Senbaru 1, Nishihara, 903-0213, Okinawa, Japan

Corresponding authors: Abdolmohammad Mehranpour (E-mail: ammehranpour@hotmail.com)

Table of content ………………………………………………………………….....

Page

Fig. S1 The ^{1}H NMR spectrum of compound **1a** ……………..………………………………S3

Fig. S2 The ^{13}C NMR spectrum of compound **1a** ……………………..…………………… S4

Fig. S3 The Mass spectrum of compound **1a** ……………………………………….……..….. S5

Fig. S4 The ^{1}H NMR spectrum of compound **1b** ……………..…………..…………………. S6

Fig. S5 The ^{13}C NMR spectrum of compound **1b** ………….……………………………….…. S7

Fig. S6 The Mass spectrum of compound **1b** ………………………………………………. S8

Fig. S7 The ^{1}H NMR spectrum of compound **1c** ……….…………………………………….. S9

Fig. S8 The ^{1}H NMR spectrum of compound **1d** ……………..……………………………….. S10

Fig. S9 The ^{1}H NMR spectrum of compound **1e** ………………………………..…………… S11

Fig. S10 The ^{1}H NMR spectrum of compound **1f** …………………………..…..…………….. S12

Fig. S11 The ^{13}C NMR spectrum of compound **1f** …………………………...…………… S13

Fig. S12 The X-RAY spectrum of compound **1f**……………………….…………..………. S14

Fig. S13 X-ray data table for compound **1f**…………………….……………….……...………. S15

Fig. S14 The High resolution Mass spectrum of compound **1f** ……………….…………….. S16

Fig. S15 The FT-IR spectrum of compound **1f** …………………………………….………… S17

Fig. S16 The Vanderwals bonds spectrum of compound **1f** …………………….………… S18

Fig. S17 The $\pi-\pi$ Bonds spectrum of compound **1f** ……………………….…………… S19

Fig. S18 The ^{1}H NMR spectrum of compound **1g** ………………………….….………….. S20

Fig. S19 The ^{13}C NMR spectrum of compound **1g** ………………………….….………… S21

Fig. S20 The ^{1}H NMR spectrum of compound **1h** ………………………….….………….. S22

Fig. S21 The ^{13}C NMR spectrum of compound **1h** ………………………….….………… S23

Fig. S22 The Mass spectrum of compound **1h** …………………………………………….. S24

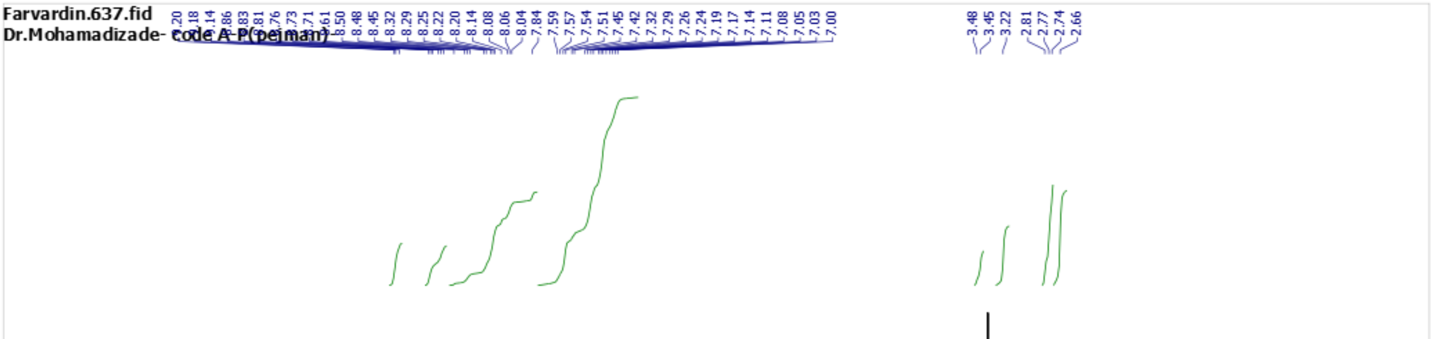

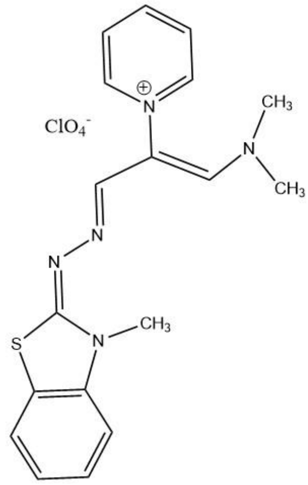

Fig. S1 The ^{1}HNMR spectrum of compound **1a**

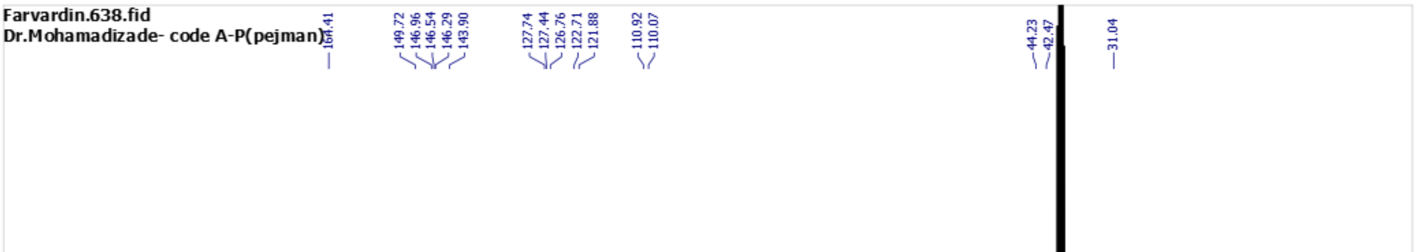

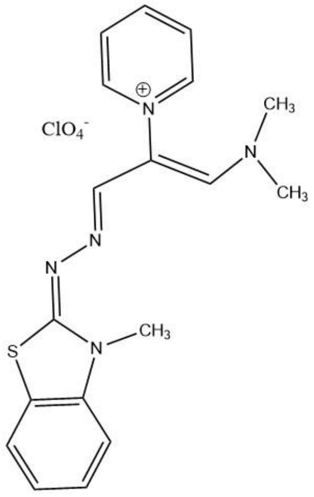

Fig. S2 The ^{13}CNMR spectrum of compound **1a**

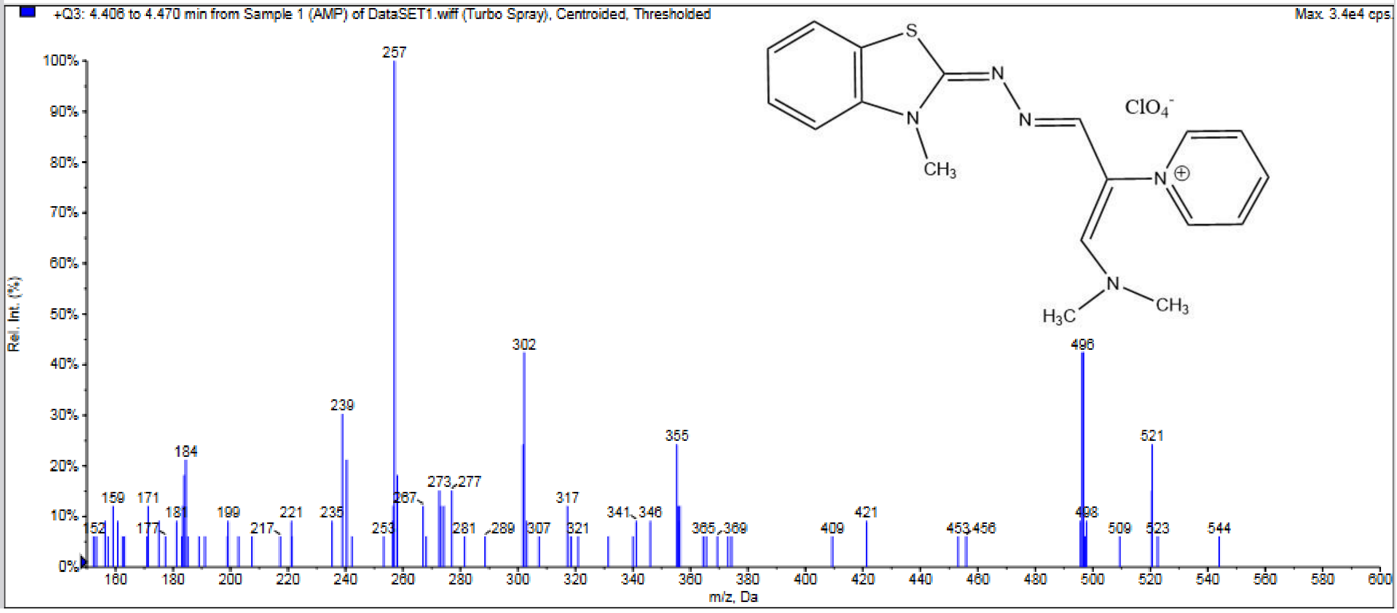

Fig. S3 The Mass spectrum of compound **1a**

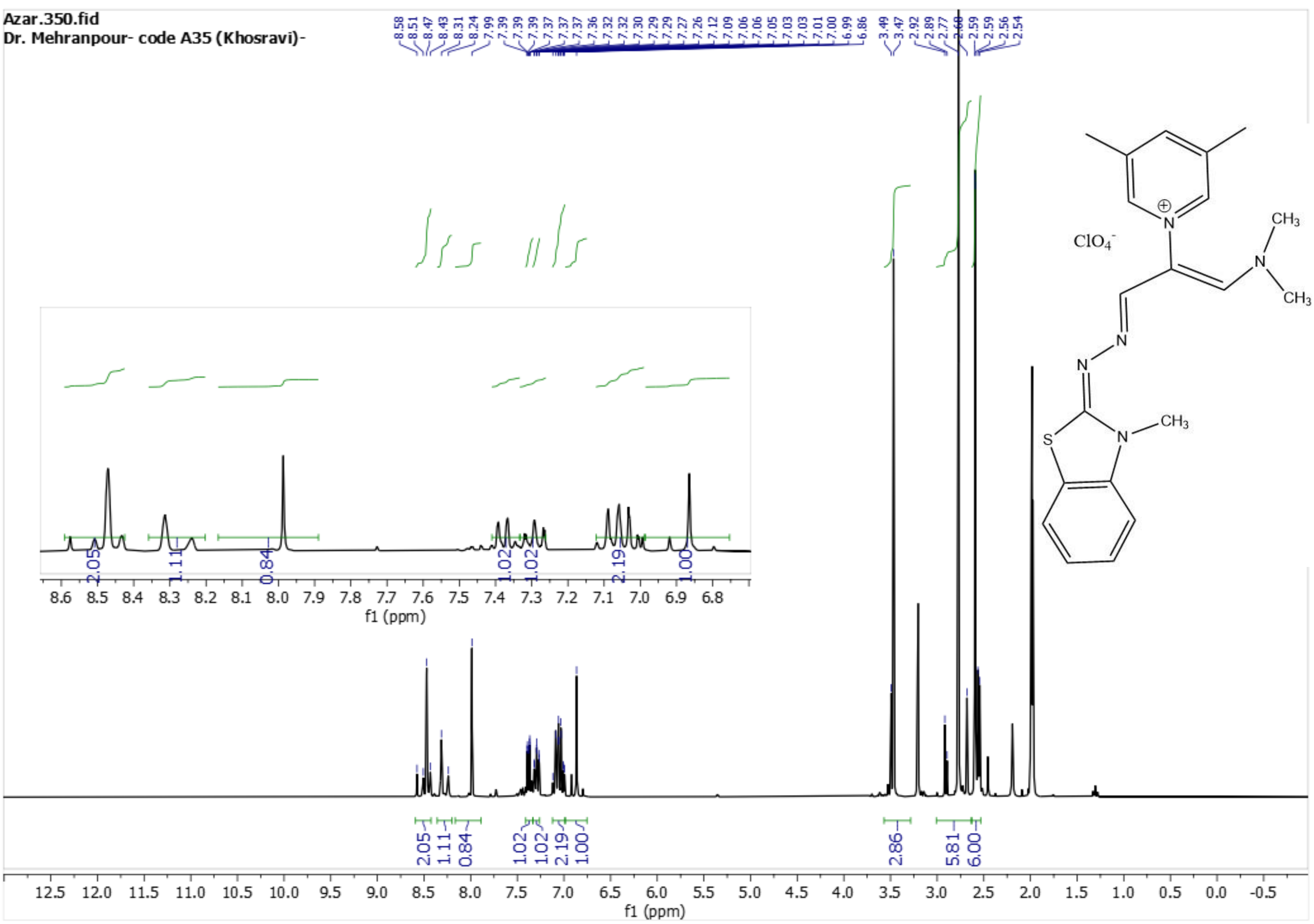

Fig. S4 The ^{1}HNMR spectrum of compound **1b**

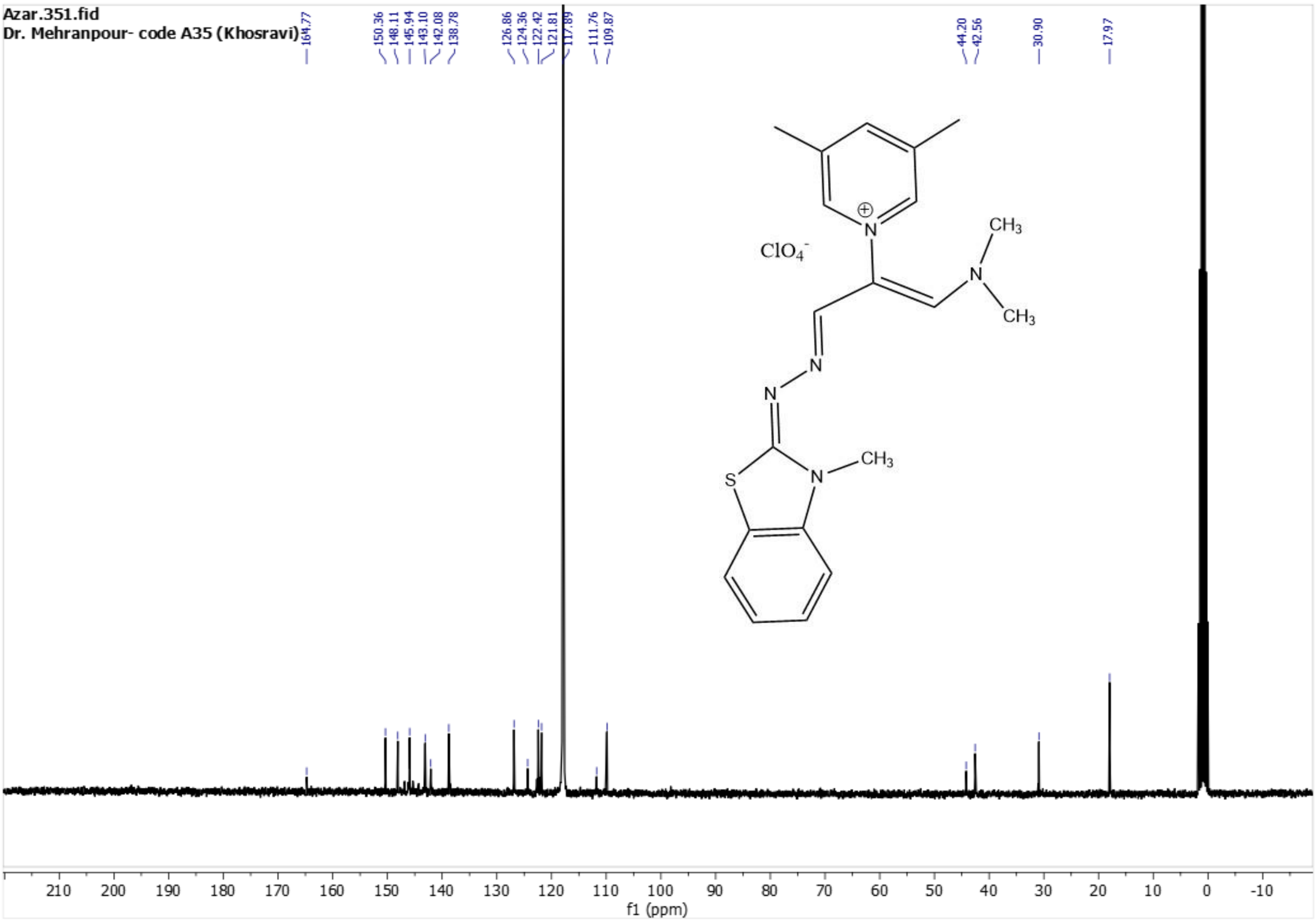

Fig. S5 The ^{13}C NMR spectrum of compound **1b**

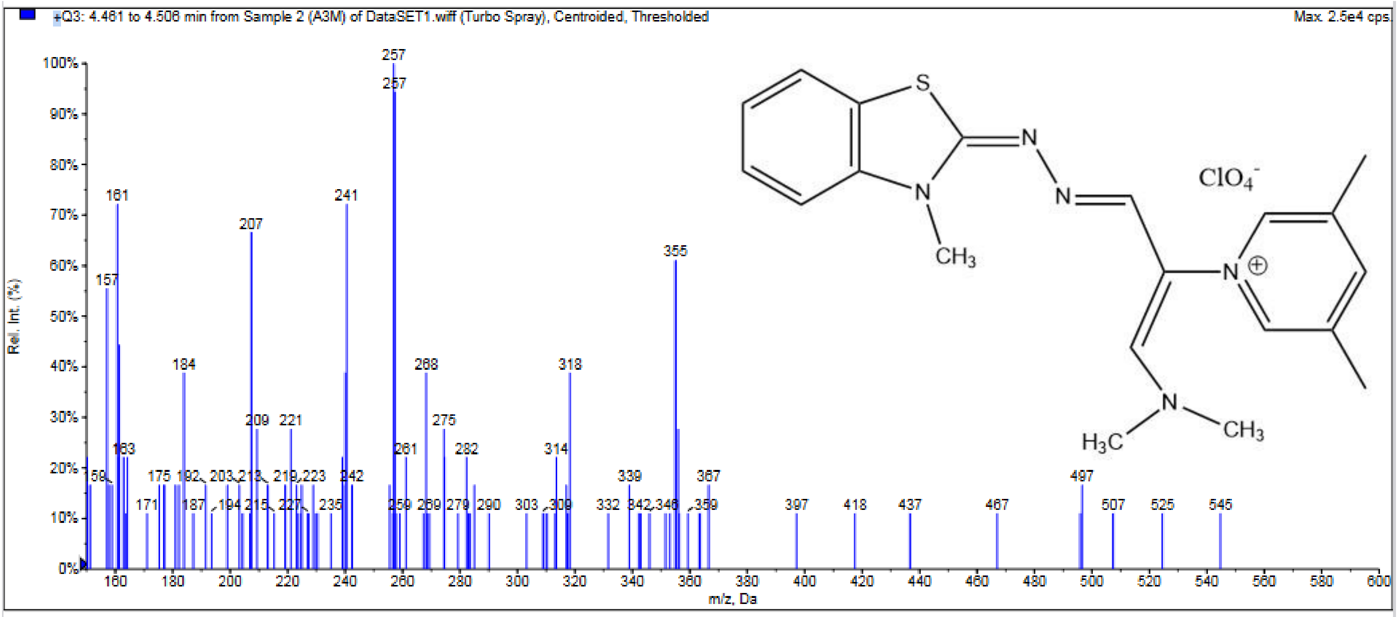

Fig. S6 The Mass spectrum of compound **1b**

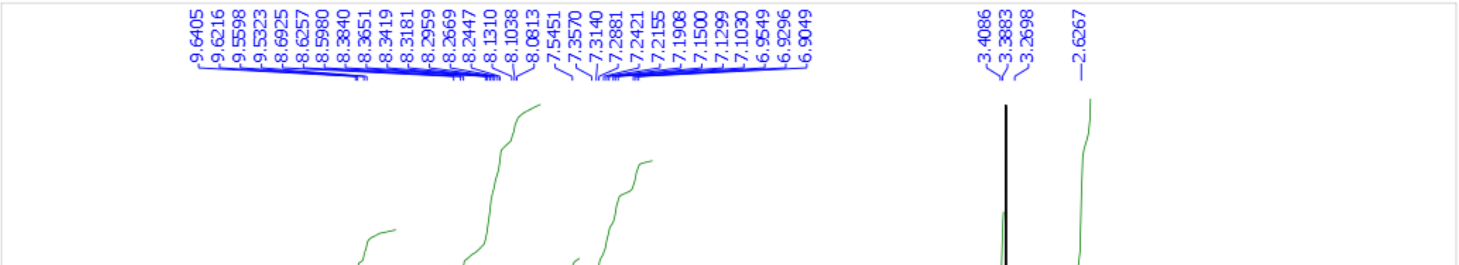

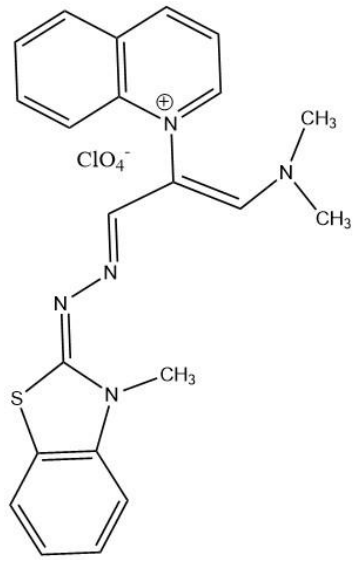

Fig. S7 The ^{1}HNMR spectrum of compound **1c**

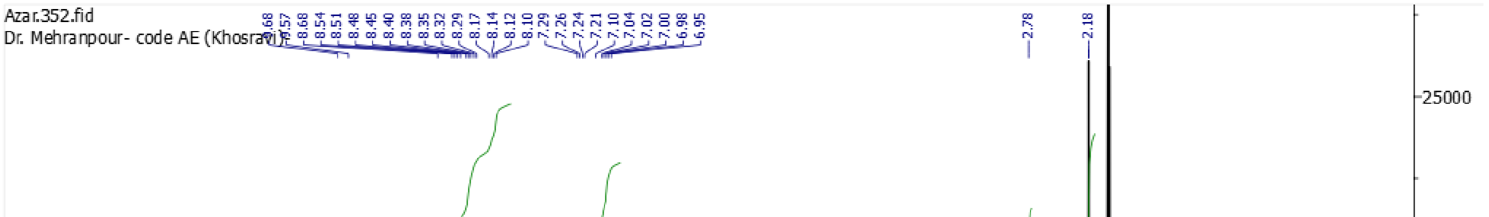

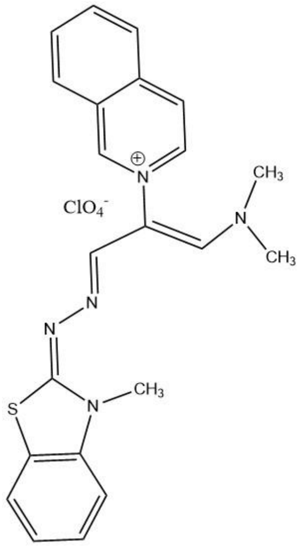

Fig. S8 The ^{1}HNMR spectrum of compound **1d**

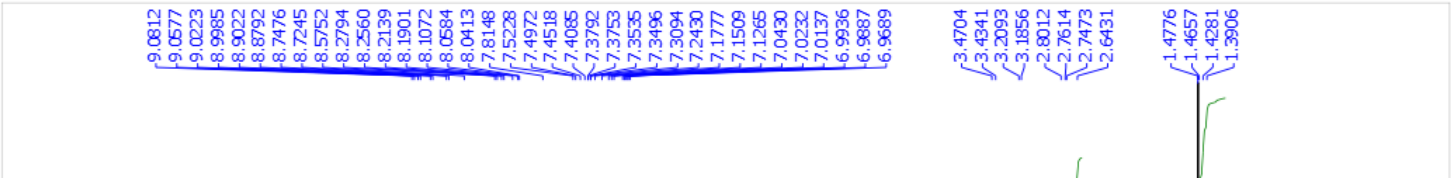

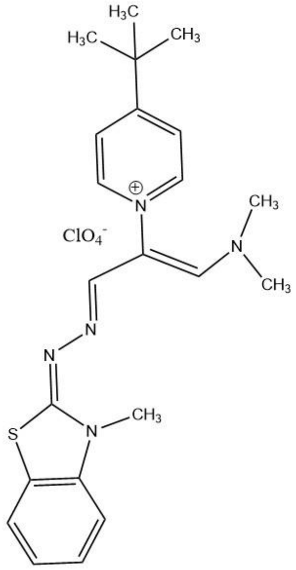

Fig. S9 The ^{1}HNMR spectrum of compound **1e**

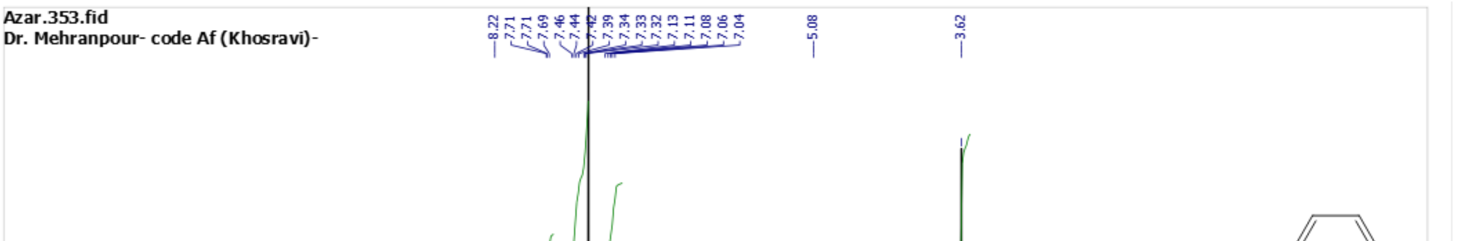

Fig. S10 The ^{1}HNMR spectrum of compound **1f**

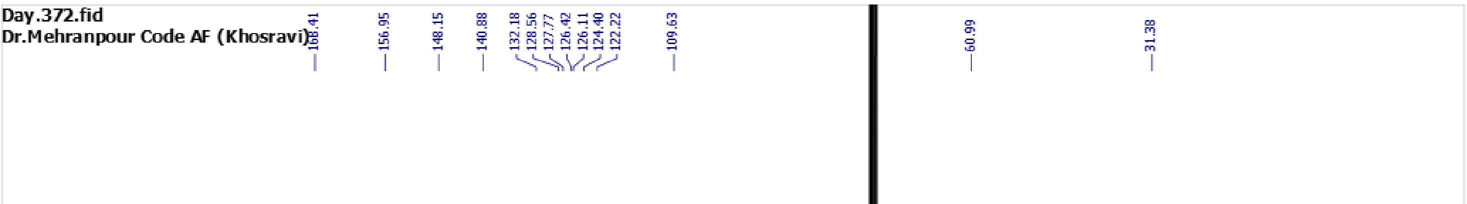

Fig. S11 The ^{13}CNMR spectrum of compound **1f**

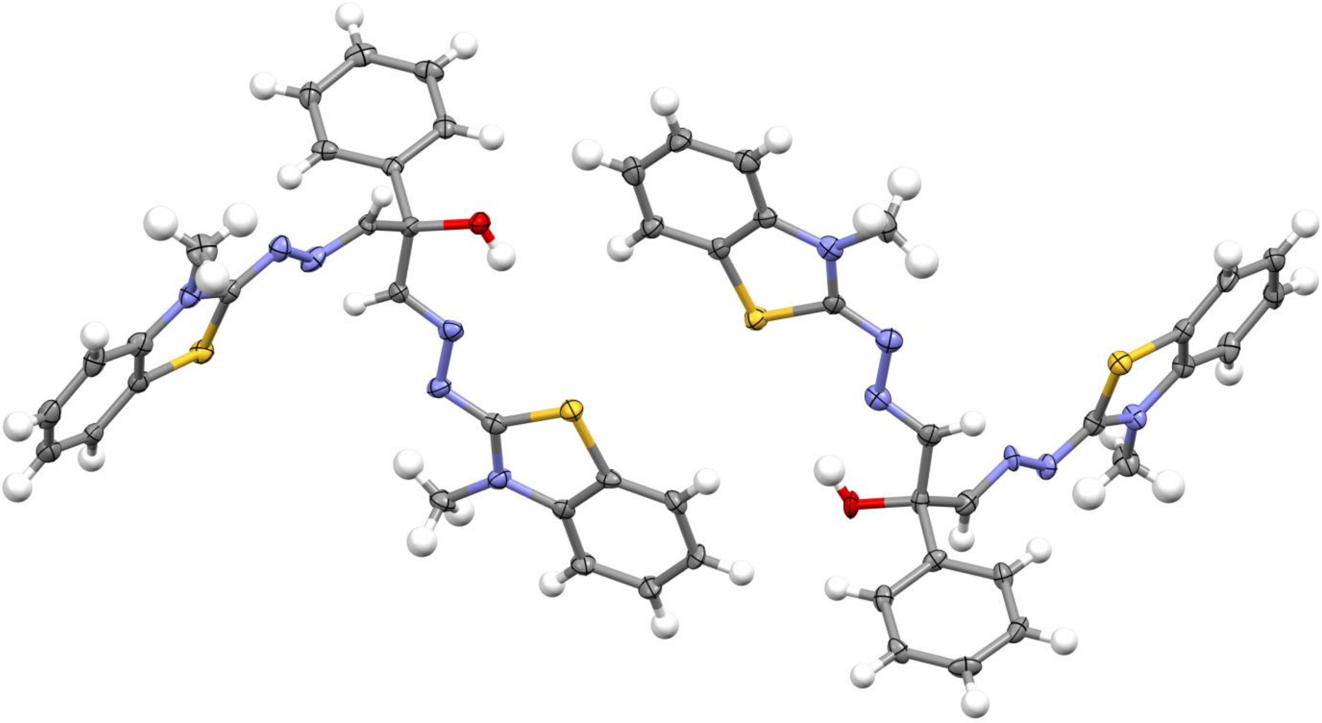

Fig. S12 The X-RAY spectrum of compound **1f**

Crystal data	
Chemical formula	2($C_{25}H_{22}N_6OS_2$)
M_r	973.21
Crystal system, space group	Orthorhombic, $Pca2_1$

Temperature (K)	103
a, b, c (Å)	26.3379 (10), 8.2678 (3), 21.3056 (7)
V (Å^3)	4639.4 (3)
Z	4
Radiation type	Mo $K\alpha$
μ (mm^{-1})	0.26
Crystal size (mm)	0.3 × 0.06 × 0.06
Data collection	
Diffractometer	Rigaku Saturn724plus
Absorption correction	Multi-scan
T_{min}, T_{max}	0.961, 1.000
No. of measured, independent and observed [$I > 2\sigma(I)$] reflections	42248, 13889, 7960
R_{int}	0.109
$(\sin \theta/\lambda)_{max}$ (Å^{-1})	0.729
Refinement	
$R[F^2 > 2\sigma(F^2)]$, $wR(F^2)$, S	0.086, 0.175, 1.03
No. of reflections	13889
No. of parameters	619
No. of restraints	1
H-atom treatment	H-atom parameters constrained
$\Delta\rho_{max}$, $\Delta\rho_{min}$ (e Å^{-3})	0.55, −0.50
Absolute structure parameter	0.45 (7)

Fig. S13 The X-RAY data of compound **1f**

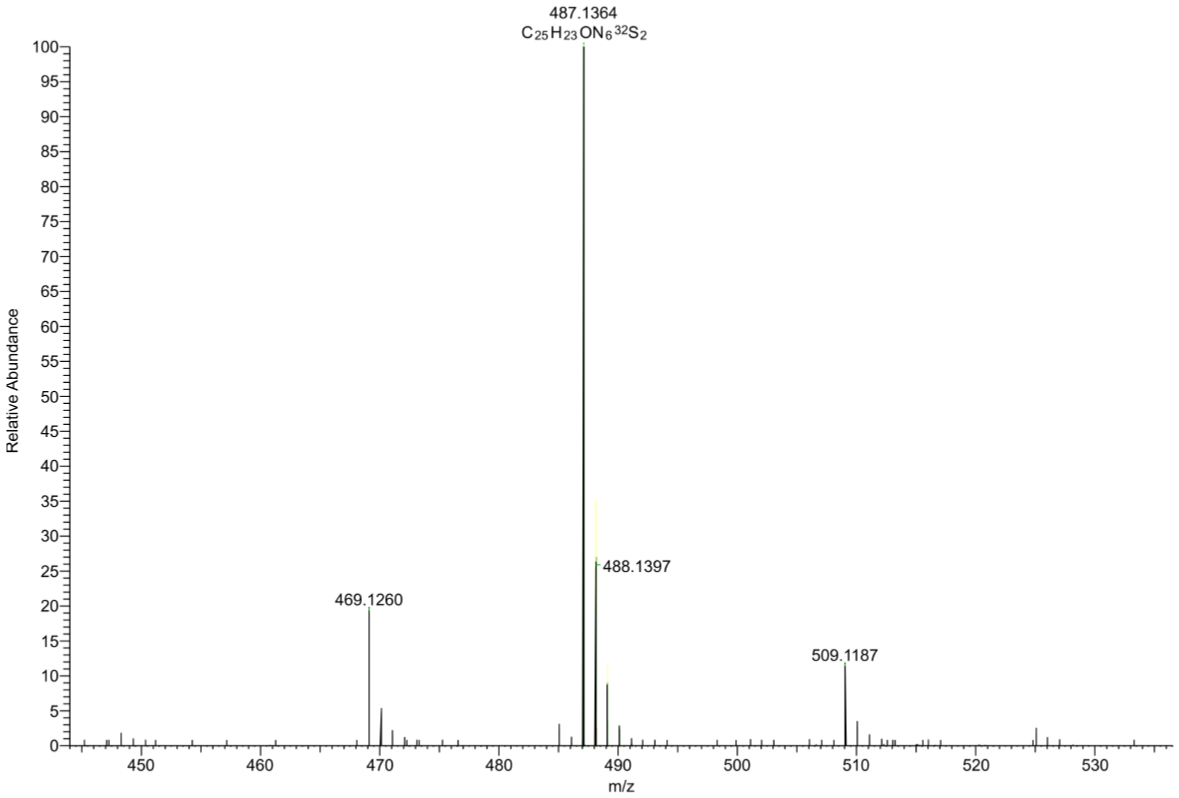

Fig. S14 The High resolution Mass spectrum of compound **1f**

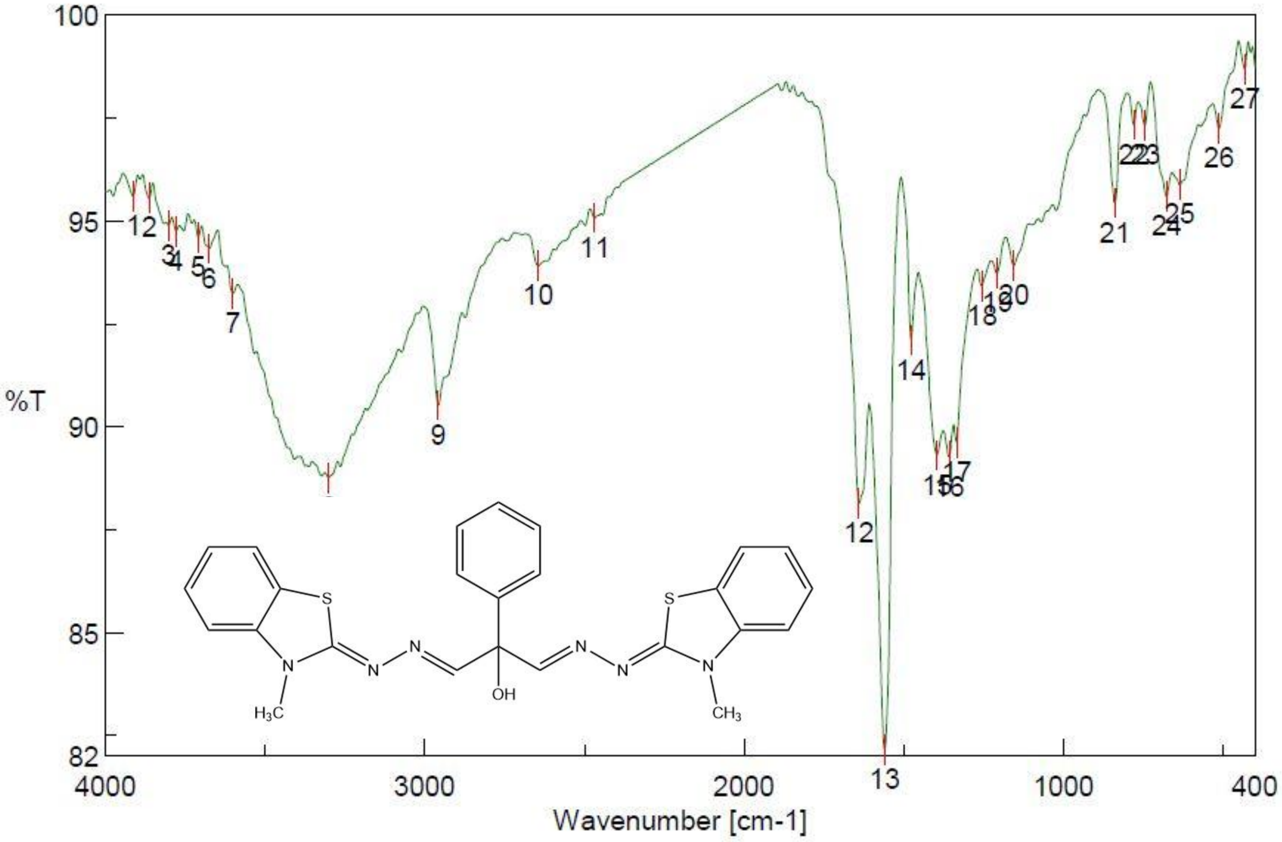

Fig. S15 The FT-IR spectrum of compound **1f**

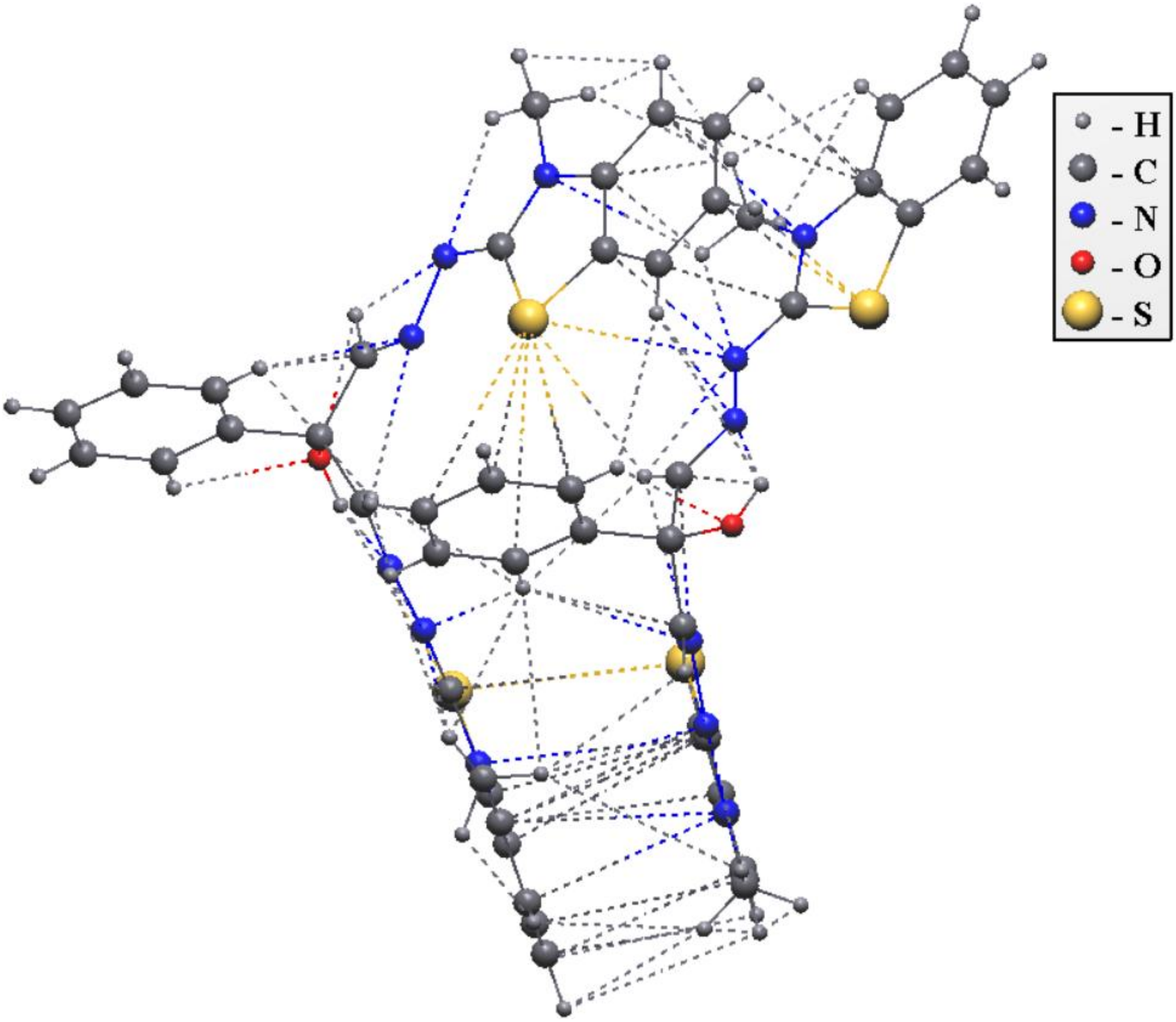

Fig. S16 The Vanderwals bonds of compound **1f**

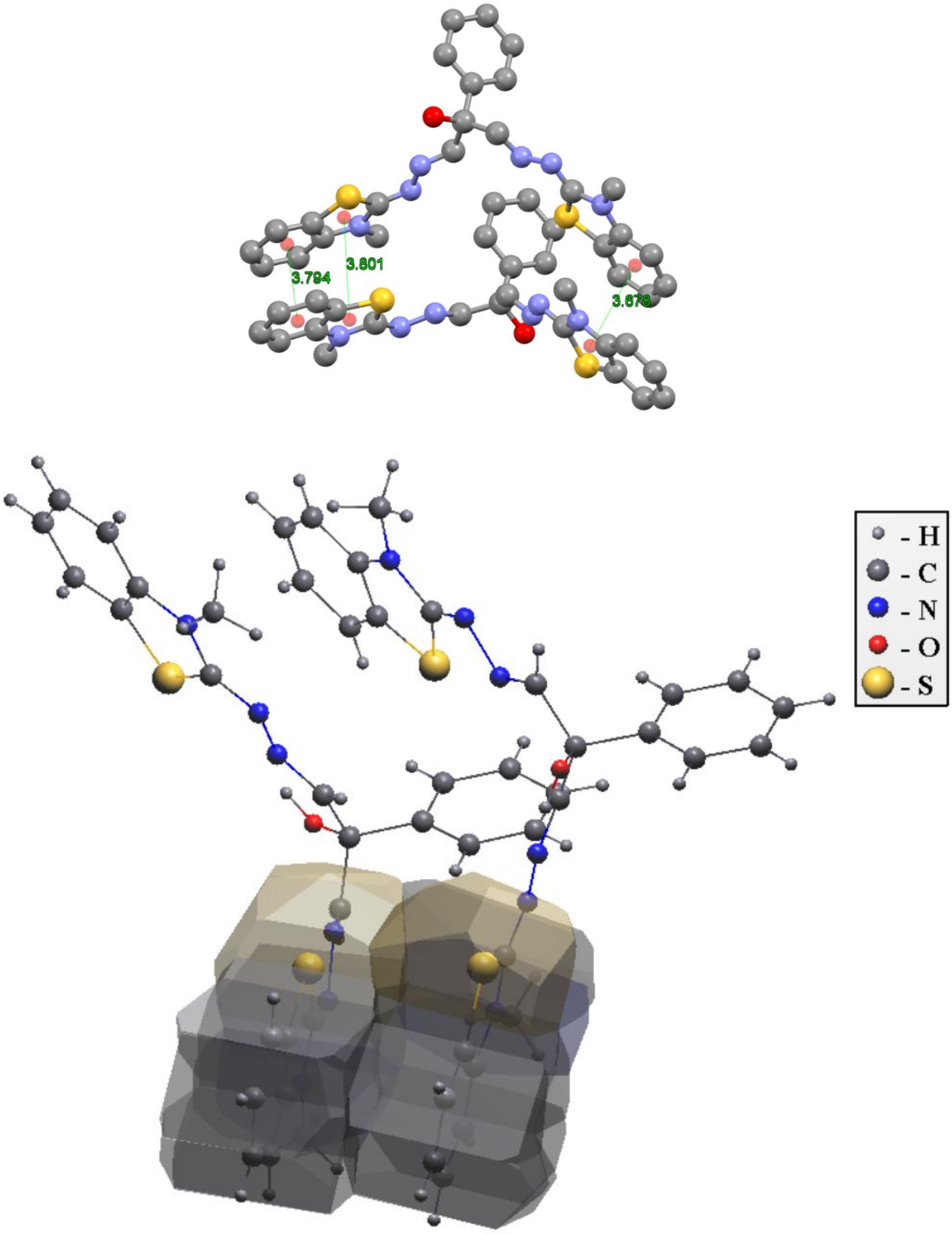

Fig. S17 The $\pi - \pi$ Bonds of compound **1f**

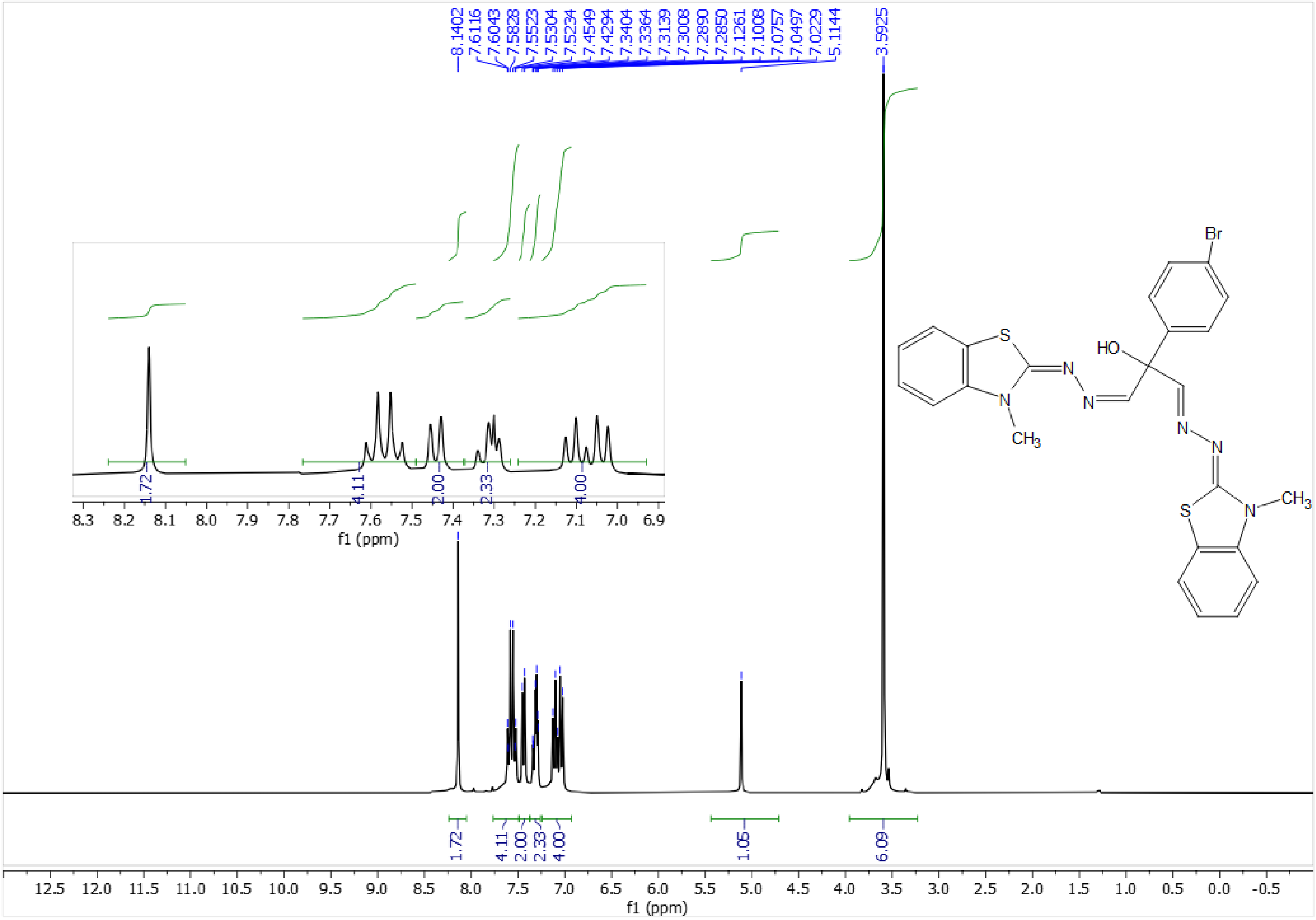

Fig. S18 The ^{1}HNMR spectrum of compound **1g**

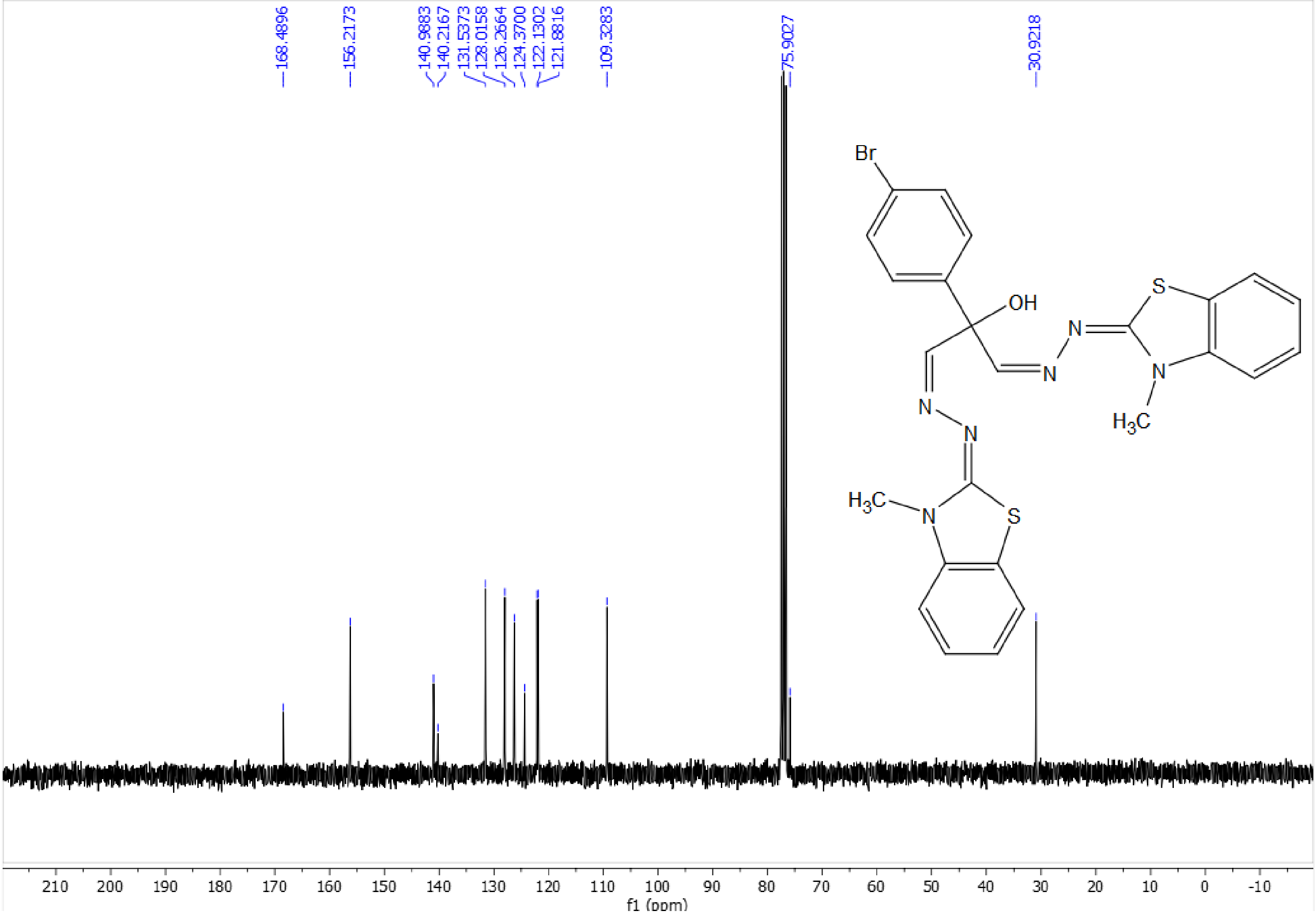

Fig. S19 The ^{13}C NMR spectrum of compound **1g**

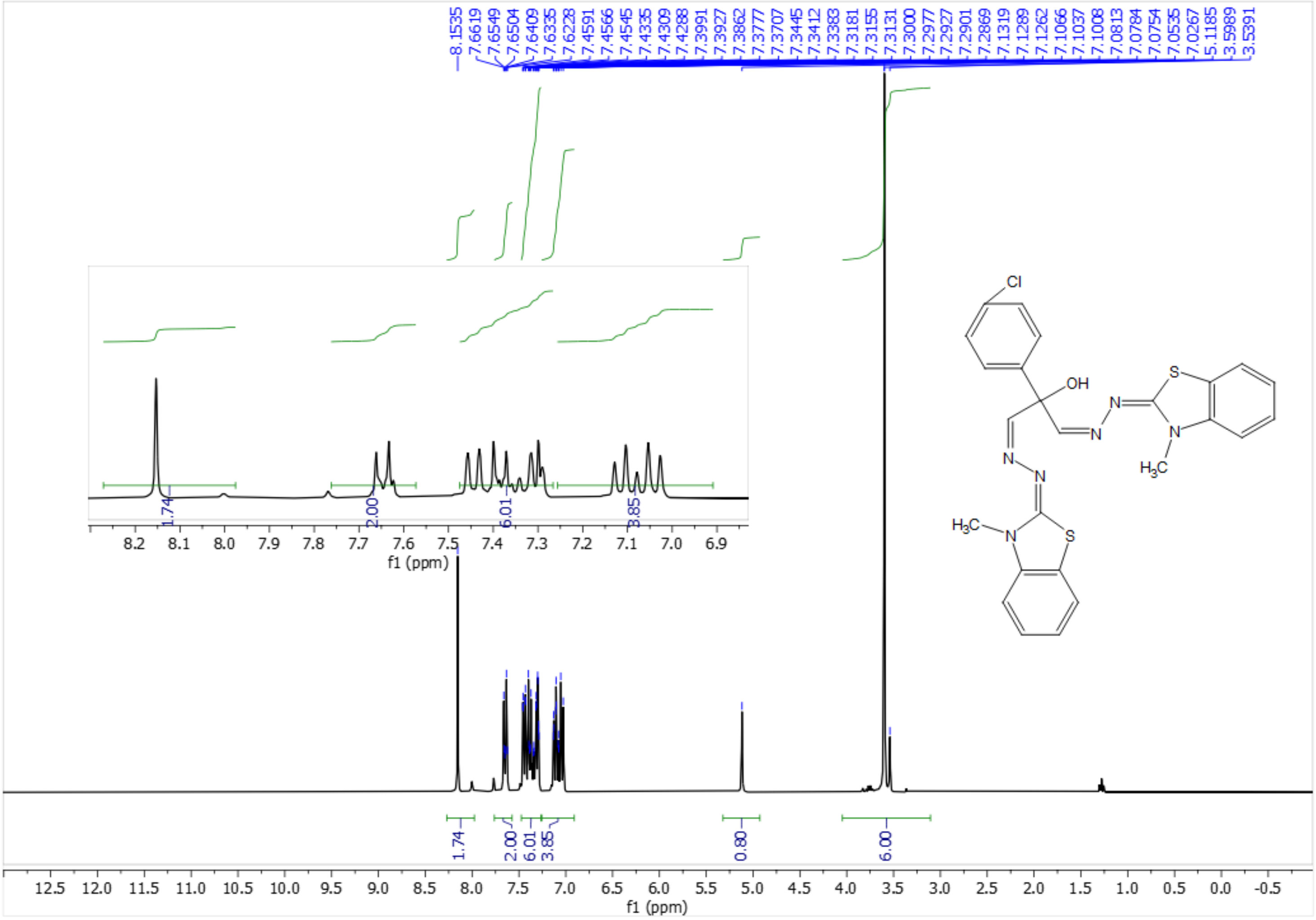

Fig. S20 The ^{1}HNMR spectrum of compound **1h**

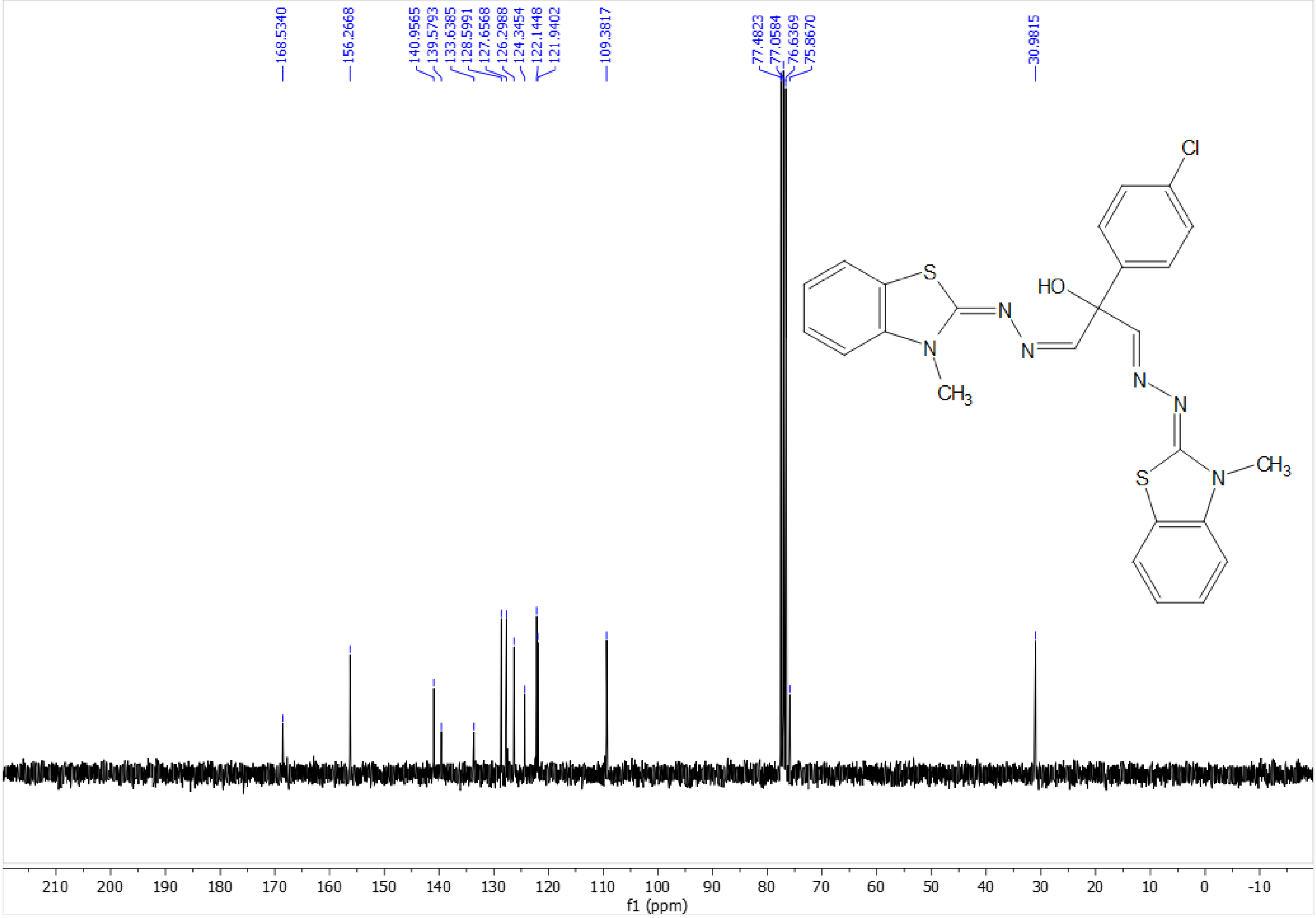

Fig. S21 The ^{13}C NMR spectrum of compound **1h**

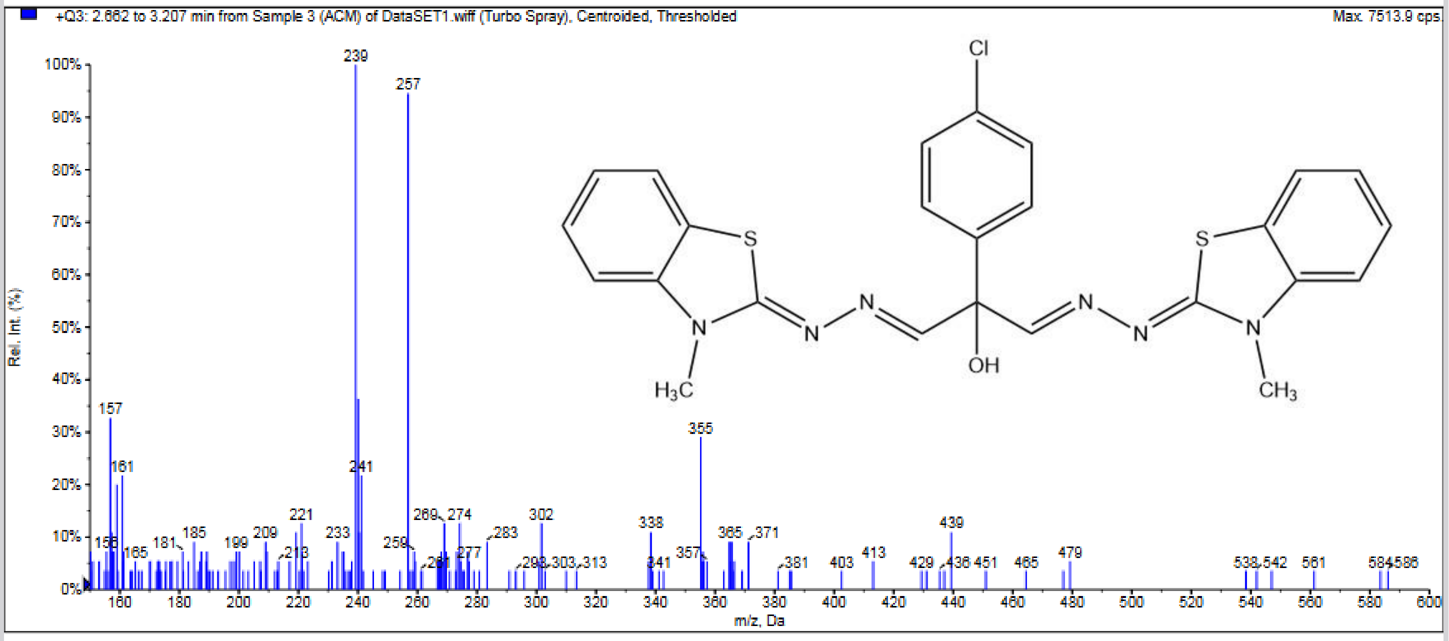

Fig. S22 The Mass spectrum of compound **1h**
